# Supplementary material for: Improving care pathways for children with severe illness through implementation of the ASPIRE mHealth primary ETAT package in Malawi
Source: PLOS Glob Public Health. 2024 Apr 29;4(4):e0002786. doi: 10.1371/journal.pgph.0002786 (PMC11057765; doi:10.1371/journal.pgph.0002786)
Supplement: S1 Fig — (DOCX) [file pgph.0002786.s002.docx]

**S1 Fig 1: mHealth primary ETAT algorithm**

1. **Child details:**
   1. Gender: <Boy/Girl>
   2. Date of birth: <date>
   3. ID number: <automatic>

***Screening***

1. **Emergency signs**
   1. Does the child have any of the following signs: <multiple select>

- Obstructed breathing
- Central cyanosis (child is blue)
- Severe respiratory distress
  - 1. If obstructed breathing or central cyanosis or severe respiratory distress, then ‘This child has an emergency sign, which requires immediate emergency treatment. Please take the child to a clinical officer immediately’.
  1. Does the child have cold hands? <single select> <y/n>
     1. If yes, then ‘Does the child have any of the following signs: <multiple select>
- Capillary refill longer than 3 seconds
- Weak and fast pulse
  - - 1. If capillary refill longer than 3 seconds and weak and fast pulse, then ‘This child has an emergency sign, which requires immediate emergency treatment. Please take the child to a clinical officer immediately’.
  1. Does the child display any of the following conditions: <multi-select>
- Coma
- Convulsions
  - - 1. If coma or convulsions, then ‘This child has an emergency sign, which requires immediate emergency treatment. Please take the child to a clinical officer immediately’.
  1. Does the child have diarrhoea?’ <single select> <y/n>
     1. If yes, then ‘Does the child have any of the following signs:’ <multiple select>
- Lethargic or unconscious
- Sunken eyes
- Very slow skin pinch
  - - 1. If (lethargic or unconscious and sunken eyes) or (lethargic or unconscious and very slow skin pinch) or (sunken eyes and very slow skin pinch), then ‘This child has an emergency sign, which requires immediate emergency treatment. Please take the child to a clinical officer immediately’.

1. **Priority signs**
   1. Does the child have any of the following signs: <multiple select>

- Infant of less than two months of age
- Temperature very high
- Trauma or other urgent surgical condition
- Severe pallor
- Poisoning
- Severe pain
- Respiratory distress
- Restless, continuously irritable, or lethargic
- Urgent referral
- Severe wasting/malnutrition
- Oedema of both feet
- Major burns
  - 1. If any of the above signs, then ‘This child has a priority sign, please take the child to the front of the queue’.

.

1. **Non-urgent cases**
   1. If any no emergency or priority signs, then ‘This child does not have any emergency or priority signs and can wait their turn in the queue’.

**Supplementary Figure 2: Prevalence-adjusted kappa values for concordance between mHealth triage and clinician assessment**
