# Supplementary material for: Improving care pathways for children with severe illness through implementation of the ASPIRE mHealth primary ETAT package in Malawi
Source: PLOS Glob Public Health. 2024 Apr 29;4(4):e0002786. doi: 10.1371/journal.pgph.0002786 (PMC11057765; doi:10.1371/journal.pgph.0002786)
Supplement: S1 Table — (DOCX) [file pgph.0002786.s003.docx]

# S1 Table 1. Topic guide for Semi-structured interviews with parents or carers

1. Can you please tell us why you came to this health facility today?

(Probe: illness & symptoms of child)

**Mungatifotokozereko chifukwa chomwe mwabwelela ku chipatala kuno**?

***(Probe: matenda omwe akudwala/ zizindikiro zimene amawonetsa)***

1. How old is the child?

**Kodi mwanayu ali ndi zaka zingati?**

1. For how long was the child ill before you came to the facility?

**Kodi mwanayi wadwala kwa nthawi yayitali bwanji musanabwele naye ku chipatala kuno?**

1. How frequently do you come to this facility?

**Kodi mumabwelabwela ku chipatala kuno?**

(Probe: when was last time they came and for what reason)

**(*Probe: ndi liti lomaliza lomwe munabwela kuno ku chipatala/ Munabwera chifukwa chani*)**

1. How did you travel to this facility today?

**Kodi lero mwayenda bwanji pobwela ku chipatala kuno?**

1. How long did it take to get to this facility?

**Kodi munayenda kwa nthawi yayitali bwanji kuti mufike ku chipatala kuno?**

1. What time did you arrive at this health facility?

**Kodi mwafika nthawi yanji ku chipatala kuno?**

1. Do you know any of the health staff at this facility? If yes, who and what is your relationship to them?

**Kodi mumadziwana ndi aliyense ogwila ntchito pa chipatala pano? Mumadziwana ndindani ndipo ubale wanu ndiwotani?**

1. Did you receive assistance from any of the staff at the facility?

(Probe: what type of assistance and by whom)

**Kodi mwalandila chithandizo kuchokela kwa anthu ogwila ntchito pa chipatala pano?**

**(Probe: chithandizo chotani ndipo wakupatsani ndani?)**

1. About how long do you think you had to wait before anyone came to help you? How did this make you feel as a client of the facility?

**Mukuganiza kuti patenga nthawi yayitali bwanji kuti muthandizidwe? Munamva bwanji podikila inu monga munthu wofuna chithandizo pa chipatala pano?**

1. Who was the first person that attended you? (HSA, nurse, clinical officer). What did they do?

**Ndindani amene anayamba kukuthandizani mutafika ku chipatala kuno? (HSA, anamwino, clinical ofisala). Anapanga chani?**

1. Can you please explain to me your overall experience today:

(Probe: communication with staff at triage area, during waiting and at consultation, understanding of what to expect and what you should do)

**Mungandifokozere zomwe mwakumana nazo lero:**

**(Probe: Kalankhulidwe ndi wogwila ntchito pa malo omwe mumadikilila kuti muwonane ndi adokotala, pomwe mumakumana ndi adokotala, Munamvetseta zomwe zimayenera kuchitika ndi zomwe mumayenera kuchita)**

1. What do you think of the quality of care/ services you received today at this facility?

**Munganenepo chani pa chisamalilo/chithandizo chomwe mwalandila ku chipatala kuno lero?**

1. If you were to compare the quality of care/services you received today at this facility to previous experiences before October, 2012 what would you say?

(Probe: communication with staff at triage area, during waiting and at consultation, understanding of what to expect and what you should do)

**Mutati musiyanitse chisamalilo/chithandizo chomwe mwalandila ku chipatala kuno lero ndi m’mbuyomu chamu Okotoba,2012 munganenepo chani?**

**(Probe: Kalankhulidwe ndi wogwila ntchito pa malo omwe mumadikilila kuti muwonane ndi adokotala, pomwe mumakumana ndi adokotala, munamvetseta zomwe zimayenera kuchitika ndi zomwe mumayenera kuchita)**

1. Have you ever heard about the chipatala robots? What is your understanding of the chipatala robots?

**Munayamba mwamvapo za chipatala loboti? kodi munamva kuti ndi chani?**

1. What colour card was your child assigned today? Did you understand why the child was assigned that color? How did it make you feel to be assigned:

**Kodi mwana wanu anapasidwa pepala lamtundu wanji lero? kodi munamvetsa chifukwa chimene mwanayo anapatsidwa mtundu umenewu? munamva bwanji mutapatsidwa:**

1. an **emergency** (seen immediately),

wodwalitsitsa (akawonedwe mwansanga)

1. **priority** (given priority in the Queue)

wodwalika (akhale kusogolo kwa nzele)

1. **Queue** (wait in the queue)?

Wodwala (adikile pa nzele)

1. What advice do you have to help us to improve patient experiences of the Chipatala Robot system?

**Mungatipatse malangizo otani omwe angathandize odwala kuti alandile chithandizo chabwino kumbali ya ndondomeko ya chipatala loboti?**

(Write down the patients PID number)
