# Supplementary material for: Improving care pathways for children with severe illness through implementation of the ASPIRE mHealth primary ETAT package in Malawi
Source: PLOS Glob Public Health. 2024 Apr 29;4(4):e0002786. doi: 10.1371/journal.pgph.0002786 (PMC11057765; doi:10.1371/journal.pgph.0002786)
Supplement: Attachment — Submitted filename: ASPIRE_overview_PLoS GPH-response.docx [file pgph.0002786.s004.docx]

Our responses to reviewer comments are below:

**Editor comments:**

- The reviewers and I agree that this manuscript addresses important questions in emergency care in Malawi, with implications for triage implementation in other resource-constrained settings.
- However, as noted by the reviewers below there are a number of areas in the methods, results, and discussion that need to be addressed. Greater clarity on the objectives of the study, the measures and methods used to assess and compare the ASPIRE approach with clinician triage, and outcomes are needed.

**Author comments:** We appreciate the comments raised by the reviewers and have noted and addressed the points raised. We have paid particular attention to clarifying the objectives and methodology.

**Reviewer #1:**

The paper is very interesting and focusses new concepts and practices in the health and medical field. The paper is good but some adjustments are needed. Please see below some of the issues that need your attention.

1. Consider adjusting the title of the paper. In its current state it does not really capture the main objective of the paper. The paper does not examine improvements on care pathways for children with severe illness but rather examines the implication of primary level mHealth ETAT package through the ASPIRE project in Malawi and how that has resulted in improved care pathways for children with severe illness in the broader sense (see line 142 – 146) . You could modify the title to read like “Implication of The ASPIRE mHealth primary ETAT approach on improving care pathways for children with severe illness in Malawi” or something along those lines that clearly elucidates the main purpose and objective of the study.

**Author comments:** We agree that the title should capture the main purpose and objective of the study. We would argue, however, that the purpose of the ASPIRE mHealth primary ETAT package is to improve care pathways for children through implementation of a package to improve triage, detection of severe illness, and referral. Therefore, we have adjusted the title of the paper but kept the key elements in the title [L1-2].

2. The paper does not have a clear study design. From lines 82-84, 91-97, 142-146 it is clear that the paper is trying to address the issue of failure to adequately optimize responses and recognition as well as management of childhood illnesses that affect quality of childhood illness care through the ASPIRE mHealth Primary ETAT system. If this is the case then, the study design should focus on this and not the implementation phases of the ASPIRE mHealth Primary ETAT system. Not that the implementation phases are not relevant, the implementation phases can be presented as part of the introduction with the study design focusing on the main approach of the study that guides the overall quantitative and qualitative analysis process of the study.

# Author comments: This work is an implementation research study, with the key focus being to develop a system integrated within the existing primary level health care in Malawi to improve care pathways for children with severe illness. Therefore, in this study, we followed the observational study design for implementation research [Hwang et al Designs and methods for implementation research: Advancing the mission of the CTSA program [J Clin Transl Sci.](https://www.ncbi.nlm.nih.gov/pmc/articles/PMC7348037/) 2020 Jun; 4(3): 159–167; Harrison MB, et al Guideline adaptation and implementation planning: a prospective observational study *Implementation Science* 2013; 8(1): 49]. We used a mixed methods approach with both quantitative and qualitative data collection and analysis with the goal of assessing and understanding the implementation context and process. We have clarified this in the methods section [L56, 161] and added more description of this in the study design [L198-204].

3. In line 400-401 it is mentioned that the study is an implementation research. Expanding or providing an elaborate justifications for the selection of this approach as well as taking into account the relevance of the various components of implementation research should suffice as an adequate study design if done within the context of the study. Consider expounding on this and use it as the overall study design.

**Author comments:** We have added a section on study design in the methods section [L198-204].

**Reviewer #2:**

This is an important paper on a critical area for primary care in Malawi, a high burden context. However the way the results are currently presented and the focus on the discussion and conclusion on successful implementation rather than the results achieved, particularly in terms of missed Emergency cases, is concerning and requires significant revisions. I have made a number of suggestions as follows.

Abstract, conclusion: is a Kappa of 0.535 “moderate concordance”? Figure 2 also indicates low concordance of emergency cases (518 mHealth, 212 clinician, and only 123 with both mHealth and clinician triage outcome as Emergency). Indeed, the emergency cases should be the focus of your results given the ETAT intervention and bad health outcomes being concentrated in emergency cases. Please report the mHealth E results in the abstract (from Figure 4, concordance is 0.31, and from Figure 5, Kappa is 0.42), and modify your conclusion accordingly both in the abstract and the main paper.

**Author comments:** The kappa rating follows the standard suggested by Cohen, who proposed that a kappa in the range of 0.21-0.40 be considered 'fair' agreement, kappa=0.41-0.60 be considered 'moderate' agreement, kappa=0.61-0.80 be considered 'substantial' agreement, and kappa >0.81 be considered 'almost perfect' agreement (Cohen J. A coefficient of agreement for nominal scales. *Educ Psychol Meas.* 1960;20:37–46). This reference is already included in the text [L311]. We have added to the text to clarify this point [L312].

Figure 4 displays the concordance proportion between mHealth triage and clinician assessment overall, which is 81·6% (127,303/ 155,931; 95% CI [81·4, 81·8]) and is correctly represented in Figure 4. Figure 5 displays the kappa statistic values for mHealth and clinician triage agreement overall, which is 0·535 (95% CI [0·530, 0·539]). These are correctly presented in the abstract [L67] and main paper [L369].

Also is Kappa appropriate given you have a gold standard (Clinician triage) – might calculating sensitivity and specificity be more appropriate?

**Author comments:** In this setting, we think clinician triage is a debatable gold standard – given that clinicians (who receive training via a 3-year BSc degree and are largely based in primary-level health facilities in Malawi) are an entity distinct from medical physicians (who complete a 5-year MBBS degree plus 18-month internship and are primarily based at secondary and tertiary facilities) and not appropriate for this study. We have therefore kept the kappa statistic.

“lower cadre health workers” – please define which cadres you mean by “lower cadre”. Perhaps ‘non-physician clinician’ would be more appropriate?

**Author comments:** In Malawi, there are clinically-trained health workers such as clinicians, nurses and medical assistants, and there are non-clinically-trained health workers such as security guards, health surveillance assistants, and other health centre staff. Given human resource shortages in this setting, non-clinically-trained health workers are involved in delivering care in primary health facilities. Therefore, in recognition of the realities of care pathways at the primary level health facilities, ASPIRE developed two manuals: one for clinically-trained, and one for non-clinically trained staff [L186-188]. Both are now Ministry-approved manuals. We have kept the phrase ‘lower cadre health workers’ but revised the text to include these clarifications [L142-145, L246].

Introduction, good background provided, though please can you explain in one sentence how exactly ASPIRE uses mHealth? this sentence near the end of the introduction could be explained: “using mHealth technology to optimise the response to severe illness in children”. Related to this the third paragraph in your background section on mHealth could more specifically cover what exactly you are doing with mHealth in ASPIRE.

**Author comments:** We have included this sentence [L157-158] and added some background on the third paragraph related mHealth and linking this to ASPIRE [L112-125-126].

Introduction, end of 4th paragraph: “However, HSAs need to have a minimum of only two years of secondary schooling, and receive only 12 weeks of training, which means that the quality of diagnostic services, especially in response to severe illness, is often suboptimal.” please provide reference(s) to back up these points.

# Author comments: We have included a reference on HSA training (Kok et al, Health surveillance assistants as intermediates between the community and health sector in Malawi: exploring how relationships influence performance BMC Health Serv Res. 2016 May 3:16:164. doi: 10.1186/s12913-016-1402-x) [L141-142]. To our knowledge, no prior publication to date has evaluated the concordance of lower cadre health workers with clinician triage. This study is the first to document this.

Methods, line 220: “triaged by mHealth” – which cadres of health worker were doing the triaging with the mHealth tool? Please make it clear that health workers were inputting data into the mHealth tool to do the triage – also how accurate was this data entry into the smart phones? In your discussion (lines 418-419) you say: “retrospective input of some data by triage staff” – this is quite concerning and suggests the triage was not always done correctly. Please explain and add an estimate to the paper of how often this may have occurred.

**Author comments:** We have clarified that all cadres of staff working in PHCs, including security guards and primarily HSAs, were conducting the triage with the mHealth tool [L254-L255].

With regards to the concern raised by the reviewer regarding the imprecise nature of triage in the study setting, we acknowledge this as a limitation. Given the management of large numbers of patients, and that the goal of this implementation study was to integrate processes into the existing health system, staff had a heavy workflow and it was not always possible for them to input data in real time. The fundamental basis of implementation research is accepting that there is a trade-off between fidelity to the protocol and the pragmatism of real world conditions. This therefore results in issues such as that described above. We have explained this further in the text [L457-458]. Given the implementation nature of this work, we do not have estimates as to how often this may have occurred.

Figure 2: please add points D1 and D2.

**Author comments:** This has been added [Fig 2].

Methods, qualitative evaluation: please add more details on your facility observations – how many observations were done? over what time period? what was recorded? and how was it analysed? and used in your study?

**Author comments:** This was an error, we had initially planned to conduct facility observations, but during implementation found that the journey modelling was sufficient to gain understanding of the layout of the primary health setting and how patients moved from one section to another. We have removed this [L422].

Methods, qualitative analysis – please add reference for the method you used. Also why did you use grounded theory rather than thematic analysis? was there no relevant prior qualitative or theoretical work you could build on? and did your qualitative analysis inform the design or implementation of your interventions at all? if so, how specifically? From the limited qualitative results presented it seems not as only positive things are mentioned and nothing about the potential for the mHealth intervention to miss emergency cases that Clinicians would catch for example, or over-diagnose emergency cases and the implications of that? Were these issued explored in your qualitative evaluation? Please add your topic guide as supplementary material. Also was your topic guide iterated at all from baseline to implementation phases based on emerging quantitative results?

**Author comments:** The methods used for the qualitative analysis are listed in the data analysis section [L320-328]. We used constant comparison approaches which are derived from grounded theory, but we used thematic content analysis and not a grounded theory approach to the analysis. Rather, in order to understand responses to the ongoing implementation programme, we triangulated across timepoints and across participants and interview methods. We removed the comment on grounded theory [L442] and included a reference for the method used.

Our qualitative analysis was done concurrently with the rest of the study, and focused solely on the acceptability and feasibility of the triage system. Therefore, interviews were conducted only at the point of first contact of the parent/guardian, and given staffing limitations, was not transcribed and analysed until the completion of the study. Therefore, this was not a two-step process where we used the results of the qualitative study to inform the design of the triage, or where we iterated the topic guide based on emerging quantitative results. The study design of interviews conducted at one point of contact meant that participants did not have follow-up interview to discuss missed cases. We had the interest and intention to conduct additional qualitative evaluations post-triage, but we were unable to secure the additional funding needed to execute this while the ASPIRE study was ongoing.

The topic guide was developed for the baseline phase. Then probing was used to understanding changes. Semi structured interviews were at the end only. FGD were at the beginning and end. We have added our topic guide as supplementary material [Supplementary Appendix 2].

Results – Figure 3 - the log scale makes it difficult to visually compare mHealth and Clinician triage outcomes. Please redo the figure as separate Figures 3A, B and C for Emergency, Priority, and Queue respectively, with mHealth and Clinician side by side for each clinic, with normal y-axis scales (not log). Could exclude “All” so the scale is not too large, and you just show for each clinic separately.

**Author comments:** In our earlier draft we actually prepared the Figure without a log scale, but on the normal y-axis scale you could not discern the number of Emergency cases because the numbers were so small. Therefore, we opted to include the log scale figure as we felt that the comparison of data was clearer visually, given the size of the patient population.

We have redone the figures as suggested above [Fig 3].

Results – Table 1 – please add columns to show Clinician results as well, so that it’s possible for the reader to compare mHealth and Clinician triage by emergency and priority sign.

**Author comments:** We have added columns for clinician triage [Table 1].

Results – Table 2 – please clarify the denominator – it’s not clear why there are 186 Emergency triaged for example, when the number of Emergency triage is 123 for both mHealth and Clinician, 212 for Clinician, and 518 for mHealth.

**Author comments:** The denominators differ based on which set of records (mHealth +/- clinician triage) we used: we had 165,694 observations with clinician triage data, and 155,931 observations with both clinician and mHealth triage data. The 212 Emergency triage is based on records with clinician triage data only (denominator 165,694), while the 186 Emergency triage is based on records with both clinician and mHealth triage data (denominator 155,931) where the clinician triaged as E. We have clarified this in the text [L318-319, 337-338] and added a footnote to Table 2.

Results: “Since kappa is independent of which triage is considered to be the reference and which the comparator, we do not distinguish between mHealth and clinician E/P/Q in this figure.” – is this appropriate given Clinician triage is your gold standard? might it be more appropriate to report sensitivity and specificity of mHealth in relation to Clinician triage? This would also enable a clearer picture to be seen of under and overdiagnosis of Emergency cases by mHealth, which should be a focus of your paper. From your discussion: “but the majority with clinician triage of E were captured by mHealth (66·1%, 123 out of 186)” it’s apparent that 34% of E cases were missed by mHealth, which is surely a major shortcoming and could have serious consequences if the mHealth intervention was more widely adopted?

**Author comments:** As stated above (p3), we did not consider clinician triage to be an appropriate gold standard and have not used this designation in our study. We could have considered physician triage a possible gold standard, but physicians are primarily based in tertiary health facilities; given that only a fraction of patients seen at the primary health centre ended up at tertiary care, we did not have the sample size to adequately evaluate this.

To focus of this paper is the implementation of ETAT are primary level to improve care pathways for children with severe illness. Therefore, the specific focus was not on under/overdiagnosis of Emergency cases by mHealth, but the improvement of care pathways in a health system with significant human resource shortage and weak infrastructure for referral of care.

As stated in the introduction, Malawi is a least developed country with an U5 mortality rate of 43 per 1,000 live births. Children are already dying, and they exist in a health system with immense resource limitations, with not least the lack of qualified, clinically-trained staff. ETAT has been demonstrated to reduce mortality, and the goal of the study was to evaluate whether primary ETAT can improve care pathways. Therefore, in this setting, we believe the findings demonstrate that that the expansion and adoption of ETAT among primary level health worker staff has the potential to identify children with severe illness, rather than as a potential major shortcoming. We have included a comment on the potential harm of missed cases [L429-433].

Discussion – as per above comments, please also discuss under- and over-diagnosis of Emergency cases by the mHealth intervention in comparison to Clinician triage, and the implications of this. Perhaps also considering the coverage of Clinician triage in routine settings and the potential for mHealth to cover more patients as a counterpoint? what might the net benefits, or harms, of the mHealth intervention be?

**Author comments:** We have included more discussion on improving care pathways for children with severe illness in a health system with immense resource limitations, and a comment on the potential harm of missed cases [L429-433].

Discussion, first paragraph: “the implementation of ETAT through a mHealth algorithm approach increased the proportion of successful referrals from primary to tertiary.” – this increase is not shown in your results – please add to your results and explain how it was calculated.

**Author comments:** We initially included these data in our results. However, these data were collected at the baseline stage before the implementation of ETAT. When we reviewed this, we did not have the rigor in our approach established at this stage to provide these details. Therefore, we considered removing the information altogether but felt there was still value in including it, albeit not in the results section. We have clarified this in the text [L460-461].

Discussion, last sentence: “we emphasise the need for additional strategies to promote sustainability, such as the development of leadership, accountability, and the strengthening of capacity at different levels of the health system.” – this sentence and this whole paragraph is written on the assumption that the intervention should continue. Should this intervention be sustained though given it could potentially lead to harm by missing Emergency cases? (see above comments)

**Author comments:** The reality in this setting is that Malawi has an existing child mortality rate of 43 per 1,000 live births: children are already dying. As stated in above (p7), in the setting of lack of human resources and trained personnel, we do believe the findings demonstrate that that the expansion and adoption of ETAT among primary level staff has the potential to identify children with severe illness. We have included a comment on the potential harm of missed cases [L429-433].

Conclusion – The conclusion is currently written as if the intervention were a success purely because it was implemented – the results of the implementation need to be the focus of your conclusion - please revise in light of the above comments, considering the potential for harm of the intervention.

**Author comments:** We have revised the conclusion to include these comments [L502-503].

**Reviewer #3:**

I am not sure if this was a study or a program. One of the major purpose and utility of a scientific article is its validity and reliability. For this, a detailed description of all variables and the tests performed needed to explain with greater detail. It is also essential to talk about the tools. For qualitative data, a detail description of the tools, purpose, themes and analysis technique is needed. The submitted manuscript lacks all of these. It is rather a detailed description of an intervention and program data in the current form. I suggest, the authors should revise it according to standard format of a scientific public health article.

**Author comments:** Please see our detailed response above (p2). Based on reviewer comments above, we have revised the manuscript, added detail to the methods section, given a detailed description of the qualitative tools and techniques used, and included a topic guide. Note that this is implementation research and therefore the format differs from the standard public health article. However, we have endeavoured to follow standard format of an observational study design reporting of implementation research [Hwang et al Designs and methods for implementation research: Advancing the mission of the CTSA program [J Clin Transl Sci.](https://www.ncbi.nlm.nih.gov/pmc/articles/PMC7348037/) 2020 Jun; 4(3): 159–167; Harrison MB, et al Guideline adaptation and implementation planning: a prospective observational study *Implementation Science* 2013; 8(1): 49].
