# Supplementary material for: Improving care pathways for children with severe illness through implementation of the ASPIRE mHealth primary ETAT package in Malawi
Source: PLOS Glob Public Health. 2024 Apr 29;4(4):e0002786. doi: 10.1371/journal.pgph.0002786 (PMC11057765; doi:10.1371/journal.pgph.0002786)
Supplement: Attachment — Submitted filename: ASPIRE_overview_PLoS GPH-response2.docx [file pgph.0002786.s005.docx]

Our responses to reviewer comments are below:

**Reviewer #1:**

All comments have been addressed.

**Author comments:** Thank you.

**Reviewer #2:**

Thanks for your revision. Unfortunately it appears to have been rushed as many of my comments from my first review were not addressed. I hope you submitted the final tracked changes version as many of the line numbers in your response have no changes. In fact as you'll see below I've stopped half way through as it seems most the revisions were not done - please check thoroughly and re-submit and I will review then.

**Author comments:** We apologise for any confusion – the tracked changes were addressed in ‘simple mark-up’ view rather than ‘all mark-up’ view and that is what led to the discrepancy in line numbering. We have reviewed the tracked changes in ‘all mark-up’ view and assigned the proper line numbering throughout. However, we do note that deleted text does not show up in the ‘all mark-up view’ and therefore there may still be some discrepancy in line numbering – we have done what we can to minimize this.

My comment about focusing on the Emergency (E) cases was not addressed: “Please report the mHealth E results in the abstract (from Figure 4, concordance is 0.31, and from Figure 5, Kappa is 0.42), and modify your conclusion accordingly both in the abstract and the main paper.”

**Author comments:** We added the sentences “Concordance for mHealth emergency triage was 0.31 with kappa 0.42” in the abstract methods [L65] and paper results [L346] and “An mHealth triage algorithm can be used by LCHWs with moderate concordance with clinician triage” in the abstract conclusion [L70-71] and discussion [L380-382].

While we take your point and considered your comment about focusing on emergency cases, the purpose of ASPIRE was to develop and evaluate whether the mHealth primary ETAT triage algorithm can be used by lower cadre health care workers and improve care pathways, and this focus on emergency triage was neither the intent nor focus of ASPIRE. Therefore, in this context, we believe the findings demonstrate that that the expansion and adoption of ETAT among primary level health worker staff has the potential to identify children with severe illness, rather than as a potential major shortcoming.Therefore, we presented the results but did not focus on the emergency results in the main paper or the abstract.

There needs to be a focus on the Emergency cases as they have the most severe outcomes i.e. getting them wrong has the most harmful consequences. Please highlight in the abstract that concordance was only 0.31 “fair” for Emergency cases, and that this has implications in that XX% of emergency cases requiring referral may be missed if mHealth triage is done instead of clinician assessment.

**Author comments:** We followed the kappa rating standard suggested by Cohen (Cohen J. A coefficient of agreement for nominal scales. *Educ Psychol Meas.* 1960;20:37–46). Kappa in the range of 0.21-0.40 was considered 'fair' agreement, kappa=0.41-0.60 was considered 'moderate' agreement, kappa=0.61-0.80 was considered 'substantial' agreement, and kappa >0.81 was considered 'almost perfect' agreement.

- We defined concordance as the proportion of triages that were identical between mHealth and clinician triage assessments [L265-266]. Kappa takes into account expected concordance, but is different from the concordance value of 0.31 stated here. 0.31 is the proportion of the 392 mHealth emergency triages that are also triaged as emergency by the clinician (0.314 = 123/492). 0.42 is the corresponding kappa (triaged as emergency by either mHealth and/or clinician). In the case of Emergency cases, Cohen’s kappa was 0.42 and therefore was interpreted as ‘moderate.’ We have therefore stated that there was “moderate concordance with clinician triage” in the abstract conclusion [L70-71], results [L346], and discussion [L380-382].

We acknowledge the reviewer’s concern about harm of missed emergency cases, but respectfully disagree that there needs to be a focus on the emergency cases. Malawi is a least developed country with an U5 mortality rate of 43 per 1,000 live births. Children are already dying, and they exist in a health system with immense resource limitations, with not least the lack of qualified, clinically-trained staff. ETAT has been demonstrated to reduce mortality, and the goal of the study was to evaluate whether primary ETAT can improve care pathways. Therefore, in this context, we believe the findings demonstrate that that the expansion and adoption of ETAT among primary level health worker staff has the potential to identify children with severe illness, rather than as a potential major shortcoming.

Acknowledging the reviewer comments, we added the sentences “We did note that the concordance between E, P, and Q were different, that mHealth appeared to err more often on the side of caution with a 31·4% (123 out of 392) concordance for E between mHealth and clinician triage, but the majority with clinician triage of E were captured by mHealth (66·1%, 123 out of 186). In our kappa-adjusted analyses, the agreement between mHealth and clinician triage of E was lower than for P and Q. In a health system with immense resource limitations, we believe the findings demonstrate that the expansion and adoption of primary-level ETAT has the potential to identify children with severe illness faster, and improve care pathways, but that this must be balanced with the potential harm of missed cases at triage.” [L400-408].

Your response below to my comment below is also not clear – there are no tracked changes in the line numbers indicated and you haven’t explained the mHealth intervention as far as I can tell – what does it add to existing prioritisation methods? – please quote the added sentences in your response. To clarify I was asking you to explain what your sentence “using mHealth technology to optimise the response to severe illness in children” means, not repeat that sentence in your paper:

**Author comments:** We explained the mHealth intervention and role in optimizing the response to severe illness in children with the sentences in the introduction: “Mobile health (mHealth) technologies, which have the capacity to overcome geographic, temporal and organisational barriers, and can promote monitoring and alerting systems, data collection, and record maintenance,^15^ have been used effectively to improve access to and quality of care in low resource health system settings.^16^ Importantly, mHealth technologies are increasingly being used to support healthcare worker (HCW) performance, from providing clinical updates, to feedback on practice, learning materials, reminders, decision-making, and adherence to standards and guidelines in care.^17^” [L102-108]. We also added the sentences “In settings where a large proportion of delivery at primary care facilities is performed by HCWs with limited exposure to formal medical training, the role of mHealth in supporting decision making may be particularly useful for resilient health system strengthening.^20^ To date, this has not been evaluated at scale in Malawi.” [L110-114].

We also added the sentence “This mHealth approach facilitated movement and faster identification of sick patients as staff were able to easily move between benches to access waiting patients in overburdened clinics.” [L214-216]

“ Introduction, good background provided, though please can you explain in one sentence how exactly ASPIRE uses mHealth? this sentence near the end of the introduction could be explained: “using mHealth technology to optimise the response to severe illness in children”. Related to this the third paragraph in your background section on mHealth could more specifically cover what exactly you are doing with mHealth in ASPIRE.

**Author comments:** We added this sentence in the introduction: “The Achieving Sustainable Primary Improvement and Engagement in health (ASPIRE) project was developed in 2013 and implemented from 2013 to 2018 to use mHealth to address primary level care and develop a sustainable approach to ETAT implementation under such resource constraints.” [L136-139] We added this sentence in the fifth paragraph rather than the third to tie together mHealth and resource constraints within the purpose of ASPIRE.

We also included the sentence “This mHealth approach facilitated movement and faster identification of sick patients as staff were able to easily move between benches to access waiting patients in overburdened clinics.” [L214-216]

Another comment that is not in the revised paper (there is no tracked changes in lines 254-255 and I searched as well is): “Author comments: We have clarified that all cadres of staff working in PHCs, including security guards and primarily HSAs, were conducting the triage with the mHealth tool [L254-L255].

**Author comments:** We added the sentences “Clinical care is supported by nursing staff, who focus primarily on maternity services at the primary level, and HSAs, who are often required to fill the gaps in service provision. HSAs need to have a minimum of only two years of secondary schooling, and receive only 12 weeks of training.^25^ Given human resource challenges, both clinically trained HCW (clinicians, nurses, medical assistants) and non-clinically trained health workers (HSAs, security guards, and other health centre staff, referred to as lower cadre health workers (LCHW)) are involved in delivering care at PHCs.” [L127-133]

Your next response is also not done in the paper, and in any case to cover all of the points in your response (copied below) would require more than one added line of text (you say L457-458):

“With regards to the concern raised by the reviewer regarding the imprecise nature of triage in the study setting, we acknowledge this as a limitation. Given the management of large numbers of patients, and that the goal of this implementation study was to integrate processes into the existing health system, staff had a heavy workflow and it was not always possible for them to input data in real time. The fundamental basis of implementation research is accepting that there is a trade-off between fidelity to the protocol and the pragmatism of real world conditions. This therefore results in issues such as that described above. We have explained this further in the text [L457-458]. Given the implementation nature of this work, we do not have estimates as to how often this may have occurred.”

**Author comments:** We expanded the limitations section to include the following sentences: “This was not a randomised controlled trial but rather implementation research that was responsive to context-driven needs. We therefore do not have control data. Also, ETAT was not operational after hours, and this may contribute to missed or incomplete patient pathways. Participants may also have skipped or bypassed steps in the patient pathway, and data recording issues in a busy clinical care setting may lead to undercounting. While Phase 1 was focused on ETAT development and data were not consistently collected during this phase, successful referral rates were below 40%. As the delivery of care was constantly evolving, we cannot claim that the increase in the proportion of successful referrals from Phase 1 to Phase 2 was solely the result of improved emergency care at PHCs. However, the data support the fact that the implementation of prioritisation, stabilisation, and treatment through a PHC treatment package was feasible and well received. As such, implementation of ETAT within the primary health system led to an increase, from Phase 1 to Phase 2, in the likelihood of those referred reaching tertiary care but rates of successful referral remained less than optimal at 54·7%. Both economic and social barriers to the referral pathway have been identified across sub-Saharan Africa including Malawi^33^ and are likely to have impacted here. While we initially aimed to measure referral times from presentation at PHC to the referral clinician through the data monitoring system, our analysis suggested potential problems with data reliability. This could have been due to internet connectivity problems, resetting of digital clocks within the phones, or retrospective input of some data by triage staff. We have thus not included times of presentation either within the PHC or on the referral pathway.” [L427-447].

I’m going to stop here as the revision needs to be thoroughly checked and re-sent, and then will require re-review.

Last sentence of conclusion needs re-writing to be clear as it’s very long, and the new additions are not clear – probably best to split into at least two sentences e.g. the original last sentence + a new additional sentence: “We suggest that supporting capacity at the primary level to stabilise and treat patients and improving pathways and linkages from primary through to tertiary for patients requiring referral, are essential components to improved health systems and require further focus, particularly in tackling dropouts along the referral pathway itself. Close attention must also be paid to potential harms of such systems, for example cases requiring referral being missed during triage.”

**Author comments:** We revised the last sentence of the conclusion to the above: “We suggest that supporting capacity at the primary level to stabilise and treat patients and improving pathways and linkages from primary through to tertiary for patients requiring referral, are essential components to improved health systems and require further focus, particularly in tackling dropouts along the referral pathway itself. Close attention must also be paid to potential harms of such systems, for example, cases requiring referral being missed during triage” [L471-477].

**Prior reviewer #2 comments:**

This is an important paper on a critical area for primary care in Malawi, a high burden context. However the way the results are currently presented and the focus on the discussion and conclusion on successful implementation rather than the results achieved, particularly in terms of missed Emergency cases, is concerning and requires significant revisions. I have made a number of suggestions as follows.

Abstract, conclusion: is a Kappa of 0.535 “moderate concordance”? Figure 2 also indicates low concordance of emergency cases (518 mHealth, 212 clinician, and only 123 with both mHealth and clinician triage outcome as Emergency). Indeed, the emergency cases should be the focus of your results given the ETAT intervention and bad health outcomes being concentrated in emergency cases. Please report the mHealth E results in the abstract (from Figure 4, concordance is 0.31, and from Figure 5, Kappa is 0.42), and modify your conclusion accordingly both in the abstract and the main paper.

**Author comments:** The kappa rating follows the standard suggested by Cohen, who proposed that a kappa in the range of 0.21-0.40 be considered 'fair' agreement, kappa=0.41-0.60 be considered 'moderate' agreement, kappa=0.61-0.80 be considered 'substantial' agreement, and kappa >0.81 be considered 'almost perfect' agreement (Cohen J. A coefficient of agreement for nominal scales. *Educ Psychol Meas.* 1960;20:37–46). This reference is already included in the text [L287]. We have added the sentence “We reported concordance based on the kappa standard suggested by Cohen^30^” to clarify this point [L287].

Figure 4 displays the concordance proportion between mHealth triage and clinician assessment overall, which is 81·6% (127,303/ 155,931; 95% CI [81·4, 81·8]) and is correctly represented in Figure 4. Figure 5 displays the kappa statistic values for mHealth and clinician triage agreement overall, which is 0·535 (95% CI [0·530, 0·539]). These data are correctly presented in the abstract [L63-64], results [L343-345], and discussion [L380-382].

We acknowledge the reviewer’s concern about harm of missed emergency cases, but respectfully disagree that there needs to be a focus on the emergency cases. Malawi is a least developed country with an U5 mortality rate of 43 per 1,000 live births. Children are already dying in a health system with immense resource limitations, with not least the lack of qualified, clinically-trained staff. ETAT has been demonstrated to reduce mortality, and the goal of the study was to evaluate whether primary ETAT can improve care pathways. Therefore, in this context, we believe the findings demonstrate that that the expansion and adoption of ETAT among primary level health worker staff has the potential to identify children with severe illness, rather than as a potential major shortcoming.

Acknowledging the reviewer comments, we added the sentences “We did note that the concordance between E, P, and Q were different, that mHealth appeared to err more often on the side of caution with a 31·4% (123 out of 392) concordance for E between mHealth and clinician triage, but the majority with clinician triage of E were captured by mHealth (66·1%, 123 out of 186). In our kappa-adjusted analyses, the agreement between mHealth and clinician triage of E was lower than for P and Q. In a health system with immense resource limitations, we believe the findings demonstrate that the expansion and adoption of primary-level ETAT has the potential to identify children with severe illness faster, and improve care pathways, but that this must be balanced with the potential harm of missed cases at triage.” [L400-408].

Also is Kappa appropriate given you have a gold standard (Clinician triage) – might calculating sensitivity and specificity be more appropriate?

**Author comments:** In this setting, we think clinician triage is a debatable gold standard – given that clinicians (who receive training via a 3-year BSc degree and are largely based in primary-level health facilities in Malawi) are an entity distinct from medical physicians (who complete a 5-year MBBS degree plus 18-month internship and are primarily based at secondary and tertiary facilities) and not appropriate for this study. We have therefore kept the kappa statistic.

“lower cadre health workers” – please define which cadres you mean by “lower cadre”. Perhaps ‘non-physician clinician’ would be more appropriate?

**Author comments:** In Malawi, there are clinically-trained health workers such as clinicians, nurses and medical assistants, and there are non-clinically-trained health workers such as security guards, health surveillance assistants, and other health centre staff. Given human resource shortages in this setting, non-clinically-trained health workers are involved in delivering care in primary health facilities. Therefore, in recognition of the realities of care pathways at the primary level health facilities, ASPIRE developed two manuals: one for clinically-trained, and one for non-clinically trained staff. This is stated in the sentences “Phase 1 (2013 to 2015) focused on the development and piloting of a digital mobile phone algorithm for primary level triage, and Phase 1b introduced a comprehensive ETAT training package for both clinicians and LCHWs in the five busiest PHCs in Blantyre District and three in Chikwawa District, including Chikwawa District Hospital.” [L190-194]. Both are now Ministry-approved manuals. We have kept the phrase ‘lower cadre health workers’ and revised the text to include these clarifications: “Clinical care is supported by nursing staff, who focus primarily on maternity services at the primary level, and HSAs, who are often required to fill the gaps in service provision. HSAs need to have a minimum of only two years of secondary schooling, and receive only 12 weeks of training.^25^ Given human resource challenges, both clinically trained HCW (clinicians, nurses, medical assistants) and non-clinically trained health workers (HSAs, security guards, and other health centre staff, referred to as lower cadre health workers (LCHW)) are involved in delivering care at PHCs.” [L127-133].

Introduction, good background provided, though please can you explain in one sentence how exactly ASPIRE uses mHealth? this sentence near the end of the introduction could be explained: “using mHealth technology to optimise the response to severe illness in children”. Related to this the third paragraph in your background section on mHealth could more specifically cover what exactly you are doing with mHealth in ASPIRE.

**Author comments:** We added this sentence in the introduction “The Achieving Sustainable Primary Improvement and Engagement in health (ASPIRE) project was developed in 2013 and implemented from 2013 to 2018 to use mHealth to address primary level care and develop a sustainable approach to ETAT implementation under such resource constraints.” [L136-139] We added this sentence in the fifth paragraph rather than the third to tie together mHealth, resource constraints, within the purpose of ASPIRE.

We also added the sentence “This mHealth approach facilitated movement and faster identification of sick patients as staff were able to easily move between benches to access waiting patients in overburdened clinics.” [L214-216]

Introduction, end of 4th paragraph: “However, HSAs need to have a minimum of only two years of secondary schooling, and receive only 12 weeks of training, which means that the quality of diagnostic services, especially in response to severe illness, is often suboptimal.” please provide reference(s) to back up these points.

# Author comments: We have included a reference on HSA training (Kok et al, Health surveillance assistants as intermediates between the community and health sector in Malawi: exploring how relationships influence performance BMC Health Serv Res. 2016 May 3:16:164. doi: 10.1186/s12913-016-1402-x) [L130]. To our knowledge, no prior publication to date has evaluated the concordance of lower cadre health workers with clinician triage. This study is the first to document this.

Methods, line 220: “triaged by mHealth” – which cadres of health worker were doing the triaging with the mHealth tool? Please make it clear that health workers were inputting data into the mHealth tool to do the triage – also how accurate was this data entry into the smart phones? In your discussion (lines 418-419) you say: “retrospective input of some data by triage staff” – this is quite concerning and suggests the triage was not always done correctly. Please explain and add an estimate to the paper of how often this may have occurred.

**Author comments:** We have clarified that all cadres of staff working in PHCs, including security guards and primarily HSAs, were conducting the triage with the mHealth tool with the sentence “Given human resource challenges, both clinically trained HCW (clinicians, nurses, medical assistants) and non-clinically trained health workers (HSAs, security guards, and other health centre staff, referred to as lower cadre health workers (LCHW)) are involved in delivering care at PHCs.” [L130-133].

With regards to the concern raised by the reviewer regarding the imprecise nature of triage in the study setting, we acknowledge this as a limitation. We expanded the limitations section to include the following sentences: “This was not a randomised controlled trial but rather implementation research that was responsive to context-driven needs. We therefore do not have control data. Also, ETAT was not operational after hours, and this may contribute to missed or incomplete patient pathways. Participants may also have skipped or bypassed steps in the patient pathway, and data recording issues in a busy clinical care setting may lead to undercounting. While Phase 1 was focused on ETAT development and data were not consistently collected during this phase, successful referral rates were below 40%. As the delivery of care was constantly evolving, we cannot claim that the increase in the proportion of successful referrals from Phase 1 to Phase 2 was solely the result of improved emergency care at PHCs. However, the data support the fact that the implementation of prioritisation, stabilisation, and treatment through a PHC treatment package was feasible and well received. As such, implementation of ETAT within the primary health system led to an increase, from Phase 1 to Phase 2, in the likelihood of those referred reaching tertiary care but rates of successful referral remained less than optimal at 54·7%. Both economic and social barriers to the referral pathway have been identified across sub-Saharan Africa including Malawi^33^ and are likely to have impacted here. While we initially aimed to measure referral times from presentation at PHC to the referral clinician through the data monitoring system, our analysis suggested potential problems with data reliability. This could have been due to internet connectivity problems, resetting of digital clocks within the phones, or retrospective input of some data by triage staff. We have thus not included times of presentation either within the PHC or on the referral pathway.” [L427-447].

Given the management of large numbers of patients, and that the goal of this implementation study was to integrate processes into the existing health system, staff had a heavy workflow and it was not always possible for them to input data in real time. Given the implementation nature of this work, we do not have estimates as to how often this may have occurred.

Figure 2: please add points D1 and D2.

**Author comments:** This has been added [Fig 2].

Methods, qualitative evaluation: please add more details on your facility observations – how many observations were done? over what time period? what was recorded? and how was it analysed? and used in your study?

**Author comments:** This was an error, we had initially planned to conduct facility observations, but during implementation found that the journey modelling was sufficient to gain understanding of the layout of the primary health setting and how patients moved from one section to another. We have removed this.

Methods, qualitative analysis – please add reference for the method you used. Also why did you use grounded theory rather than thematic analysis? was there no relevant prior qualitative or theoretical work you could build on? and did your qualitative analysis inform the design or implementation of your interventions at all? if so, how specifically? From the limited qualitative results presented it seems not as only positive things are mentioned and nothing about the potential for the mHealth intervention to miss emergency cases that Clinicians would catch for example, or over-diagnose emergency cases and the implications of that? Were these issued explored in your qualitative evaluation? Please add your topic guide as supplementary material. Also was your topic guide iterated at all from baseline to implementation phases based on emerging quantitative results?

**Author comments:** The methods used for the qualitative analysis are listed in the data analysis section [L295-302]. We used constant comparison approaches which are derived from grounded theory, but we used thematic content analysis and not a grounded theory approach to the analysis. Rather, in order to understand responses to the ongoing implementation programme, we triangulated across timepoints and across participants and interview methods. We removed the comment on grounded theory.

Our qualitative analysis was done concurrently with the rest of the study, and focused solely on the acceptability and feasibility of the triage system. Therefore, interviews were conducted only at the point of first contact of the parent/guardian, and given staffing limitations, was not transcribed and analysed until the completion of the study. Therefore, this was not a two-step process where we used the results of the qualitative study to inform the design of the triage, or where we iterated the topic guide based on emerging quantitative results. The study design of interviews conducted at one point of contact meant that participants did not have follow-up interview to discuss missed cases. We had the interest and intention to conduct additional qualitative evaluations post-triage, but we were unable to secure the additional funding needed to execute this while the ASPIRE study was ongoing.

The topic guide was developed for the baseline phase. Then probing was used to understanding changes. Semi structured interviews were conducted at the end only, and focus group discussions were held at the beginning and end. We have added our topic guide as supplementary material [Supplementary Appendix 2].

Results – Figure 3 - the log scale makes it difficult to visually compare mHealth and Clinician triage outcomes. Please redo the figure as separate Figures 3A, B and C for Emergency, Priority, and Queue respectively, with mHealth and Clinician side by side for each clinic, with normal y-axis scales (not log). Could exclude “All” so the scale is not too large, and you just show for each clinic separately.

**Author comments:** We have redone the figures as suggested above [Fig 3].

Results – Table 1 – please add columns to show Clinician results as well, so that it’s possible for the reader to compare mHealth and Clinician triage by emergency and priority sign.

**Author comments:** We have added columns for clinician triage [Table 1].

Results – Table 2 – please clarify the denominator – it’s not clear why there are 186 Emergency triaged for example, when the number of Emergency triage is 123 for both mHealth and Clinician, 212 for Clinician, and 518 for mHealth.

**Author comments:** The denominators differ based on which set of records we used (mHealth +/- clinician triage): we had 165,694 observations with clinician triage data, and 155,931 observations with both clinician and mHealth triage data. The 212 Emergency triage is based on records with clinician triage data only (denominator 165,694), while the 186 Emergency triage is based on records with both clinician and mHealth triage data (denominator 155,931) where the clinician triaged as E. We have clarified this in the text with the sentences “For frequency of emergency signs, tabulated numbers are conditional on mHealth triage data having been recorded (denominator 195,151) whereas for referral data, numbers are conditioned on both mHealth and clinician triage (denominator 155,931).” [291-293] and added a footnote to Table 2 [L340-341].

Results: “Since kappa is independent of which triage is considered to be the reference and which the comparator, we do not distinguish between mHealth and clinician E/P/Q in this figure.” – is this appropriate given Clinician triage is your gold standard? might it be more appropriate to report sensitivity and specificity of mHealth in relation to Clinician triage? This would also enable a clearer picture to be seen of under and overdiagnosis of Emergency cases by mHealth, which should be a focus of your paper. From your discussion: “but the majority with clinician triage of E were captured by mHealth (66·1%, 123 out of 186)” it’s apparent that 34% of E cases were missed by mHealth, which is surely a major shortcoming and could have serious consequences if the mHealth intervention was more widely adopted?

**Author comments:** As stated above (p5), we did not consider clinician triage to be an appropriate gold standard and have not used this designation in our study. We could have considered physician triage a possible gold standard, but physicians are primarily based in tertiary health facilities; given that only a fraction of patients seen at the primary health centre ended up at tertiary care, we did not have the sample size to adequately evaluate this.

To focus of this paper is the implementation of ETAT are primary level to improve care pathways for children with severe illness. Therefore, the specific focus was not on under/overdiagnosis of Emergency cases by mHealth, but the improvement of care pathways in a health system with significant human resource shortage and weak infrastructure for referral of care.

As stated in the introduction, Malawi is a least developed country with an U5 mortality rate of 43 per 1,000 live births. Children are already dying, and they exist in a health system with immense resource limitations, with not least the lack of qualified, clinically-trained staff. ETAT has been demonstrated to reduce mortality, and the goal of the study was to evaluate whether primary ETAT can improve care pathways. Therefore, in this setting, we believe the findings demonstrate that that the expansion and adoption of ETAT among primary level health worker staff has the potential to identify children with severe illness, rather than as a potential major shortcoming. We have included the sentence “In a health system with immense resource limitations, we believe the findings demonstrate that the expansion and adoption of primary-level ETAT has the potential to identify children with severe illness faster, and improve care pathways, but that this must be balanced with the potential harm of missed cases at triage.” [L404-408].

Discussion – as per above comments, please also discuss under- and over-diagnosis of Emergency cases by the mHealth intervention in comparison to Clinician triage, and the implications of this. Perhaps also considering the coverage of Clinician triage in routine settings and the potential for mHealth to cover more patients as a counterpoint? what might the net benefits, or harms, of the mHealth intervention be?

**Author comments:** Please see our response above. We have included the sentence “In a health system with immense resource limitations, we believe the findings demonstrate that the expansion and adoption of primary-level ETAT has the potential to identify children with severe illness faster, and improve care pathways, but that this must be balanced with the potential harm of missed cases at triage.” [L404-408].

Discussion, first paragraph: “the implementation of ETAT through a mHealth algorithm approach increased the proportion of successful referrals from primary to tertiary.” – this increase is not shown in your results – please add to your results and explain how it was calculated.

**Author comments:** We initially included these data in our results. However, these data were collected at the baseline stage before the implementation of ETAT. When we reviewed this, we did not have the rigor in our approach established at this stage to provide these details. Therefore, we considered removing the information altogether but felt there was still value in including it, albeit not in the results section. We have removed this sentence from the first paragraph of the discussion and added the sentence “While Phase 1 was focused on ETAT development and data were not consistently collected during this phase, successful referral rates were below 40%.” [L432-433].

Discussion, last sentence: “we emphasise the need for additional strategies to promote sustainability, such as the development of leadership, accountability, and the strengthening of capacity at different levels of the health system.” – this sentence and this whole paragraph is written on the assumption that the intervention should continue. Should this intervention be sustained though given it could potentially lead to harm by missing Emergency cases? (see above comments)

**Author comments:** The reality in this setting is that Malawi has an existing child mortality rate of 43 per 1,000 live births: children are already dying. As stated in above (p7), in the setting of lack of human resources and trained personnel and high U5 mortality, we do believe the findings demonstrate that that the expansion and adoption of ETAT among primary level staff has the potential to identify children with severe illness. We have included the sentence “In a health system with immense resource limitations, we believe the findings demonstrate that the expansion and adoption of primary-level ETAT has the potential to identify children with severe illness faster, and improve care pathways, but that this must be balanced with the potential harm of missed cases at triage.” [L404-408].

Conclusion – The conclusion is currently written as if the intervention were a success purely because it was implemented – the results of the implementation need to be the focus of your conclusion - please revise in light of the above comments, considering the potential for harm of the intervention.

**Author comments:** We have revised the conclusion to include the sentences “The aim from the outset was to ensure a sustainable implementation approach that did not require the recruitment of additional staff but drew on the skills and needs of those working within the government health system. Despite these constraints, the system was responsive and functioned well, highlighting its utility for HCWs of all cadres in these settings. That only half of referred patients reached the tertiary site indicates an opportunity for interventions to close the referral gap. We suggest that supporting capacity at the primary level to stabilise and treat patients and improving pathways and linkages from primary through to tertiary for patients requiring referral, are essential components to improved health systems and require further focus, particularly in tackling dropouts along the referral pathway itself. Close attention must also be paid to potential harms of such systems, for example cases requiring referral being missed during triage.” [L466-477].
