# Supplementary material for: Improving care pathways for children with severe illness through implementation of the ASPIRE mHealth primary ETAT package in Malawi
Source: PLOS Glob Public Health. 2024 Apr 29;4(4):e0002786. doi: 10.1371/journal.pgph.0002786 (PMC11057765; doi:10.1371/journal.pgph.0002786)
Supplement: Attachment — Submitted filename: ASPIRE_overview_PLoS GPH-response3.docx [file pgph.0002786.s006.docx]

Our responses to reviewer comments are below:

**Editor comments:**

- The reviewer and I thank the authors for making their revisions clearer. The manuscript is much-improved, but there are few areas where additional edits are needed before this manuscript can be accepted..
- In addition to the wording suggestions below, please pay special attention to Reviewer 2's question concerning the emergency triage aspect of the ETAT tool (points 2 and 3 below).

**Author comments:** Thank you. Please note our responses to Reviewer 2 below.

**Reviewer #2:**

Thanks for clearly showing all tracked changes and for your additional revisions to the paper, which is much improved. I still have a few comments below related to prior points and your revisions.

1. Line 399-400 “mHealth appeared to err more often on the side of caution with 31.4% (123 out of 390) concordance between mHealth and clinician triage”. Should the end of this sentence say “between clinician triage and mHealth” to make it clear that the 123 is the clinician triage and 390 is the mHealth? Or even say explicitly that the 123 is clinician triage and the 390 mHealth? Otherwise it could be interpreted as mHealth identifying 123 of the 390 identified by clinicians.

**Author comments:** We edited the sentence to read: “We did note that the concordance between E, P, and Q were different, that mHealth appeared to err more often on the side of caution with 31·4% mHealth E triages confirmed by a clinician (specifically, out of 392 mHealth E triages, 123 were confirmed by the clinician triage), but the majority of clinician E triages captured by mHealth (66·1%, 123 out of 186).” [L398-400]

2. Author response: “While we take your point and considered your comment about focusing on emergency cases, the purpose of ASPIRE was to develop and evaluate whether the mHealth primary ETAT triage algorithm can be used by lower cadre health care workers and improve care pathways, and this focus on emergency triage was neither the intent nor focus of ASPIRE”
Given ETAT is Emergency Triage Assessment and Treatment, why is “this focus on
emergency triage [i.e. the ET in ETAT] neither the intent or focus of ASPIRE?” ?

**Author comments:** Thanks for the comment and we take the reviewer’s point – and would like to remove the part of the sentence where we state this.

3. Following from point 2 the above, I still think the emergency results should be a focus in the main paper, abstract, and conclusion. Also because it is the emergency cases that contribute the most to the bottom line of poor health outcomes and mortality, which as you explain in your introduction section should be the main focus of interventions in high-mortality contexts like Malawi. It still remains unclear to me from the results in your paper that expanding ETAT to lower-cadre health workers using mHealth would have net benefits on mortality in Malawi (I’m also saying this as someone who has been involved in child health research in Malawi for 17 years). Although Clinicians with 3 year BSc training may not be gold standards, they are surely more accurate than the lower-cadre health workers using the mHealth.

**Author comments:** We have added focus on the emergency results. In low resource settings where there is very little clinical capacity, the focus is on stabilization for emergency cases, followed by referral pathways so that sick patients can be transferred to higher level care in a timely manner. We have added this sentence to the main paper [L437-439].

The fact that there were a higher number of emergency cases identified by LCHW through the mHealth algorithm indicates the greater likelihood of timely referral and appropriate treatment. One of the stated goals of ASPIRE was to improve prioritization of severe illness. We have added this comment in the conclusion [L468-470] and abstract [L58].

We note that the mHealth implementation component of ETAT is designed to identify emergency and priority symptoms to get the patient to a clinician sooner than they would have if they didn’t have mHealth. Therefore, the goal is not about upskilling LCHW to be as accurate as clinicians, but to over-identify priority cases for sick patient to be seen sooner. The study was not designed to assess net benefits on mortality.

4. Author comments: We have clarified that all cadres of staff working in PHCs, including security guards and primarily HSAs, were conducting the triage with the mHealth tool with the sentence “Given human resource challenges, both clinically trained HCW (clinicians, nurses, medical assistants) and non-clinically trained health workers (HSAs, security guards, and other health centre staff, referred to as lower cadre health workers (LCHW)) are involved in delivering care at PHCs.” [L130-133].
Please change the end of this sentence from “are involved in delivering care in PHCs” to “were conducting triage with the mHealth tool” to clarify this.

**Author comments:** Change made [p6].
